# Supplementary material for: Coarsened Data in Small Area Estimation: A Bayesian Two‐Part Model for Mapping Smoking Behavior
Source: Stat Med. 2026 Oct 14;45(23-24):e70780. doi: 10.1002/sim.70780 (PMC13669427; doi:10.1002/sim.70780)
Supplement: Supplementary file 1 [file SIM-45-0-s001.pdf]

# Supplementary material of: Coarsened data in small area estimation: a Bayesian two-part model for mapping smoking behaviour

Aldo Gardini, Lorenzo Mori

This Supplementary Material provides additional theoretical, methodological, and empirical results that support the analysis presented in the main paper. Section S1 reports the formal proof of Proposition 1, while Section S2 presents the proof of Proposition 2, focusing on the existence of posterior moments of the proposed indicators. Additional simulation results assessing the performance of both model-based and design-based estimators are reported in Section S3. Finally, Section S4 contains complementary application results, including the assessment of the algorithm convergence, posterior predictive checks and model coefficients.

## S1 Proof of Proposition 1

The expected value of a generic replicate $\tilde{z}_{id}|\boldsymbol{\theta}$, considering a covariate pattern $\tilde{\mathbf{x}}_{id}$, is:

$$\mathbb{E}\left[\tilde{z}_{id}|\boldsymbol{\theta}\right] = \pi(\tilde{\mathbf{x}}_{id}, \boldsymbol{\theta}_\pi) \exp\left\{r\tilde{\mu}_{1,id} + \frac{r^2}{2}\sigma_1^2\right\} + \left[1 - \pi(\tilde{\mathbf{x}}_{id}, \boldsymbol{\theta}_\pi)\right] \exp\left\{r\tilde{\mu}_{2,id} + \frac{r^2}{2}\sigma_2^2\right\},$$

where $\tilde{\mu}_{l_{id},id} = \beta^{\mu}_{0l_{id}} + \tilde{\mathbf{x}}_{id}^{\top}\boldsymbol{\beta}^{\mu} + u^{\mu}_{d}$. Our target quantity is the $r$-th moment of the posterior predictive distribution:

$$\mathbb{E}\left[\tilde{z}^r_{id}|\mathbf{z}^\star_s, \mathbf{h}_s\right] = \int_{\boldsymbol{\Theta}} \mathbb{E}\left[\tilde{z}_{id}|\boldsymbol{\theta}\right] p(\boldsymbol{\theta}|\mathbf{z}^\star_s, \mathbf{h}_s)\mathrm{d}\boldsymbol{\theta}. \tag{S1}$$

Focusing on the posterior $p(\boldsymbol{\theta}|\mathbf{z}^\star_s, \mathbf{h}_s)\mathrm{d}\boldsymbol{\theta}$, we can express it as:

$$\begin{aligned} p(\boldsymbol{\theta}|\mathbf{z}^\star_s, \mathbf{h}_s)\mathrm{d}\boldsymbol{\theta} &= k(\mathbf{z}^\star_s, \mathbf{h}_s, \boldsymbol{\gamma}) \left[\prod_{\forall(i,d)} f_{Z^\star,H}(z^\star_{id}, h_{id}|\boldsymbol{\theta}, \boldsymbol{\gamma})\right] p(\boldsymbol{\theta}) \\ &\leq k(\mathbf{z}^\star_s, \mathbf{h}_s, \boldsymbol{\gamma}) p(\boldsymbol{\theta}), \end{aligned}$$

as each likelihood term defined as in (8) is bounded above by 1, noting that $k(\mathbf{z}^\star_s, \mathbf{h}_s, \boldsymbol{\gamma})$ denotes a term that is constant with respect to $\boldsymbol{\theta}$.

It is possible to use this upper bound for the posterior distribution in order to obtain an upper bound for the $r$-th moment:

$$\mathbb{E}\left[\tilde{z}^r_{id}|\mathbf{z}^\star_s, \mathbf{h}_s\right] \leq k(\mathbf{z}^\star_s, \mathbf{h}_s, \boldsymbol{\gamma}) \int_{\boldsymbol{\Theta}} \mathbb{E}\left[\tilde{z}_{id}|\boldsymbol{\theta}\right] p(\boldsymbol{\theta})\mathrm{d}\boldsymbol{\theta}.$$

Introducing now the expression of the $r$-th moment under the LNM model we can split the integral in two terms

$$\mathbb{E}\left[\tilde{z}_{id}^r|\mathbf{z}_s^\star,\mathbf{h}_s\right] \leq k(\mathbf{z}_s^\star,\mathbf{h}_s,\boldsymbol{\gamma})\Bigg[\int_{\boldsymbol{\Theta}_1}\pi(\tilde{\mathbf{x}}_{id},\boldsymbol{\theta}_\pi)\exp\left\{r\tilde{\mu}_{1,id}+\frac{r^2}{2}\sigma_1^2\right\}p(\boldsymbol{\theta}_1)\mathrm{d}\boldsymbol{\theta}_1+ \int_{\boldsymbol{\Theta}_2}\left[1-\pi(\tilde{\mathbf{x}}_{id},\boldsymbol{\theta}_\pi)\right]\exp\left\{r\tilde{\mu}_{2,id}+\frac{r^2}{2}\sigma_2^2\right\}p(\boldsymbol{\theta}_2)\mathrm{d}\boldsymbol{\theta}_2\Bigg];$$

where $\boldsymbol{\theta}_1$ and $\boldsymbol{\theta}_2$ contain the parameters involved in the first and the second integrals, respectively.

Given that $\pi(\tilde{\mathbf{x}}_{id},\boldsymbol{\theta}_\pi)<1,\ \forall\boldsymbol{\theta}_\pi$, we can now provide a new upper bound for the integral, integrating out $\boldsymbol{\theta}_\pi$

$$\mathbb{E}\left[\tilde{z}_{id}^r|\mathbf{z}_s^\star,\mathbf{h}_s\right] \leq k(\mathbf{z}_s^\star,\mathbf{h}_s,\boldsymbol{\gamma})\Bigg[\int_{\boldsymbol{\Theta}_1'}\exp\left\{r\tilde{\mu}_{1,id}+\frac{r^2}{2}\sigma_1^2\right\}p(\boldsymbol{\theta}_1')\mathrm{d}\boldsymbol{\theta}_1'+ \int_{\boldsymbol{\Theta}_2'}\exp\left\{r\tilde{\mu}_{2,id}+\frac{r^2}{2}\sigma_2^2\right\}p(\boldsymbol{\theta}_2')\mathrm{d}\boldsymbol{\theta}_2'\Bigg];$$

where $\boldsymbol{\theta}_1'=\boldsymbol{\theta}_1\setminus\boldsymbol{\theta}_\pi$ and $\boldsymbol{\theta}_2'=\boldsymbol{\theta}_2\setminus\boldsymbol{\theta}_\pi$.

Remarking that $p(\boldsymbol{\theta}_l')=p(\beta_{0l}^\mu)p(\boldsymbol{\beta}^\mu)p(u_d|\tau_\mu)p(\tau_\mu)p(\sigma_l)$, and recalling that $p(\beta_{0l}^\mu)$ and $p(\boldsymbol{\beta}^\mu)$ are Gaussian priors with known parameters and that $u_d^\mu|\tau_\mu\sim\mathcal{N}(0,\tau_\mu^2)$, it is possible to integrate them out, noting that we basically have a sequence of moment generating functions of Gaussian random variables:

$$\mathbb{E}\left[\tilde{z}_{id}^r|\mathbf{z}_s^\star,\mathbf{h}_s\right] \leq k'(\mathbf{z}_s^\star,\mathbf{h}_s,\boldsymbol{\gamma})\Bigg[\int_{\mathbb{R}^+\times\mathbb{R}^+}\exp\left\{\frac{r^2}{2}\sigma_1^2\right\}\exp\left\{\frac{r^2}{2}\tau_\mu^2\right\}p(\tau_\mu)p(\sigma_1)\mathrm{d}\tau_\mu\mathrm{d}\sigma_1+ \int_{\mathbb{R}^+\times\mathbb{R}^+}\exp\left\{\frac{r^2}{2}\sigma_2^2\right\}\exp\left\{\frac{r^2}{2}\tau_\mu^2\right\}p(\tau_\mu)p(\sigma_2)\mathrm{d}\tau_\mu\mathrm{d}\sigma_2\Bigg].$$

To conclude the proof, it is possible to notice that the two integrals are finite if and only if the priors of $\sigma_1$, $\sigma_2$, and $\tau_\mu$ contain terms that let the integrands go to 0 when $\sigma_1\to+\infty$,$\sigma_2\to+\infty$, and $\tau_\mu\to+\infty$.

# S2 Proof of Proposition 2

The first part of the proof concerns the fact that both $\overline{HS}_d$ and $\bar{z}_d$ are ratios of random variables, requiring additional steps to prove the existence of moments. In particular, the Holder inequality and the reverse Holder inequality can be applied in order to show the existence conditions for their $r$-th posterior moment, following parallel steps to those in Gardini et al. (2022).

Let us consider the generic indicator $\zeta_d=\frac{\zeta_{\mathrm{NUM},d}}{\zeta_{\mathrm{DEN},d}}$, then we can apply the Holder inequality with $p=1$ and $q=\infty$ obtaining:

$$\mathbb{E}\left[\zeta_d^r|\mathbf{w}_s,\mathbf{z}_s^\star,\mathbf{h}_s\right]\leq\left\|\frac{1}{\zeta_{\mathrm{DEN},d}^r}|\mathbf{w}_s,\mathbf{z}_s^\star,\mathbf{h}_s\right\|_\infty\mathbb{E}\left[\zeta_{\mathrm{NUM},d}^r|\mathbf{w}_s,\mathbf{z}_s^\star,\mathbf{h}_s\right]$$

Due to assumption (17), i.e. that the denominator is strictly positive, it holds:

$$\exists\varepsilon>0 \text{ s.t. } \mathbb{P}\left[\zeta_{\mathrm{DEN},d}>\varepsilon|\mathbf{w}_s\right]=1\implies\exists M=\varepsilon^{-r}<\infty \text{ s.t. } \mathbb{P}\left[\zeta_{\mathrm{DEN},d}^{-r}<M|\mathbf{w}_s\right]=1,$$

from which it follows that

$$\left\| \frac{1}{\zeta^{r}_{\text{DEN},d}} | \mathbf{w}_s, \mathbf{z}^{\star}_s, \mathbf{h}_s \right\|_{\infty} < \infty;$$

and, consequently, $\mathbb{E}\left[\zeta^r_d | \mathbf{w}_s, \mathbf{z}^{\star}_s, \mathbf{h}_s\right] < \infty$ if $\mathbb{E}\left[\zeta^r_{\text{NUM},d} | \mathbf{w}_s, \mathbf{z}^{\star}_s, \mathbf{h}_s\right] < \infty$.

On the other hand, by applying the reverse Holder inequality we obtain that

$$\mathbb{E}\left[\zeta_d | \mathbf{w}_s, \mathbf{z}^{\star}_s, \mathbf{h}_s\right] \geq \mathbb{E}\left[\zeta^{r/U}_{\text{NUM},d} | \mathbf{w}_s, \mathbf{z}^{\star}_s, \mathbf{h}_s\right]^{U} \mathbb{E}\left[\zeta^{r|1-U|}_{\text{DEN},d} | \mathbf{w}_s, \mathbf{z}^{\star}_s, \mathbf{h}_s\right]^{1-U}, \; U > 1.$$

From the assumption of strict positivity of the denominator, we can again state that

$$\mathbb{E}\left[\zeta^{r|1-U|}_{\text{DEN},d} | \mathbf{w}_s, \mathbf{z}^{\star}_s, \mathbf{h}_s\right]^{1-U} < \infty.$$

Consequently, $\mathbb{E}\left[\zeta_d | \mathbf{w}_s, \mathbf{z}^{\star}_s, \mathbf{h}_s\right] < \infty$ only if $\mathbb{E}\left[\zeta^r_{\text{NUM},d} | \mathbf{w}_s, \mathbf{z}^{\star}_s, \mathbf{h}_s\right] < \infty$, letting $U \to 1$.

Focusing on the existence of the moments at the numerator, we now start from $\mathbb{E}\left[\overline{HS}^r_{\text{NUM},d} | \mathbf{w}_s, \mathbf{z}^{\star}_s, \mathbf{h}_s\right]$. It is a linear combination of moments of the indicator functions $\mathbf{1}_{[20,\infty)}\left(\widetilde{w}_{id}\tilde{z}_{id}\right)$ up to the order $r$, which are finite as they constitute a bounded function. For this reason $\mathbb{E}\left[\overline{HS}^r_d | \mathbf{w}_s, \mathbf{z}^{\star}_s, \mathbf{h}_s\right] < \infty$ under any proper prior choice. Conversely, $\mathbb{E}\left[\bar{z}^r_{\text{NUM},d} | \mathbf{w}_s, \mathbf{z}^{\star}_s, \mathbf{h}_s\right]$ can be expressed as a combination of moments of the predictive distribution up to the $r$-th order. For this reason, $\mathbb{E}\left[\bar{z}^r_d | \mathbf{w}_s, \mathbf{z}^{\star}_s, \mathbf{h}_s\right] < \infty$ when $\mathbb{E}\left[\tilde{z}^r_{id} | \mathbf{z}^{\star}_s, \mathbf{h}_s\right] < \infty$.

# S3 Additional simulation results

Before to move to the additional simulation results let us briefly introduce the design-based estimators (see, among others, Wu and Thompson, 2020).

The quantities $\bar{z}_d$ and $\overline{HS}_d$ can be estimated using design-based (i.e., direct) estimators that rely solely on the sampling information. We denote by $\iota_{id}$ the first-order inclusion probability of unit $i$ in area $d$, with the estimator defined as follows:

- the average daily number of cigarette smoked by daily smokers:

$$\widehat{\bar{z}}_d = \frac{\sum_{i=1}^{n_d} \iota_{id}^{-1} w_{id} \cdot z_{id}}{\sum_{i=1}^{n_d} \iota_{id}^{-1} w_{id}}, \qquad \text{assuming} \sum_{i=1}^{n_d} \iota_{id}^{-1} w_{id} > 0;$$

- the proportion of heavy smokers, i.e. those smoking more than 20 cigarettes per day:

$$\widehat{\overline{HS}}_d = \frac{\sum_{i=1}^{n_d} \iota_{id}^{-1} \mathbf{1}_{[20,\infty)}\left(w_{id} z_{id}\right)}{\sum_{i=1}^{n_d} \iota_{id}^{-1} w_{id}}, \qquad \text{assuming} \sum_{i=1}^{n_d} \iota_{id}^{-1} w_{id} > 0.$$

Table S1 reports average performance measures for the design-based estimators under four different heaping scenarios, with the first column corresponding to the case in which no heaping is applied to the sample. While the direct estimator of $\bar{z}_d$ remains approximately unbiased, the estimator of $\overline{HS}_d$ exhibits increasing bias and variability as the severity of heaping increases, highlighting its sensitivity to data coarsening.

Figure S1 compares the relative bias and the average width of the 90% credible intervals for model-based estimators across the four simulation scenarios. The proposed model (LNM-C) display lower bias and tighter intervals, particularly for $\overline{HS}_d$ and in settings with stronger heaping.

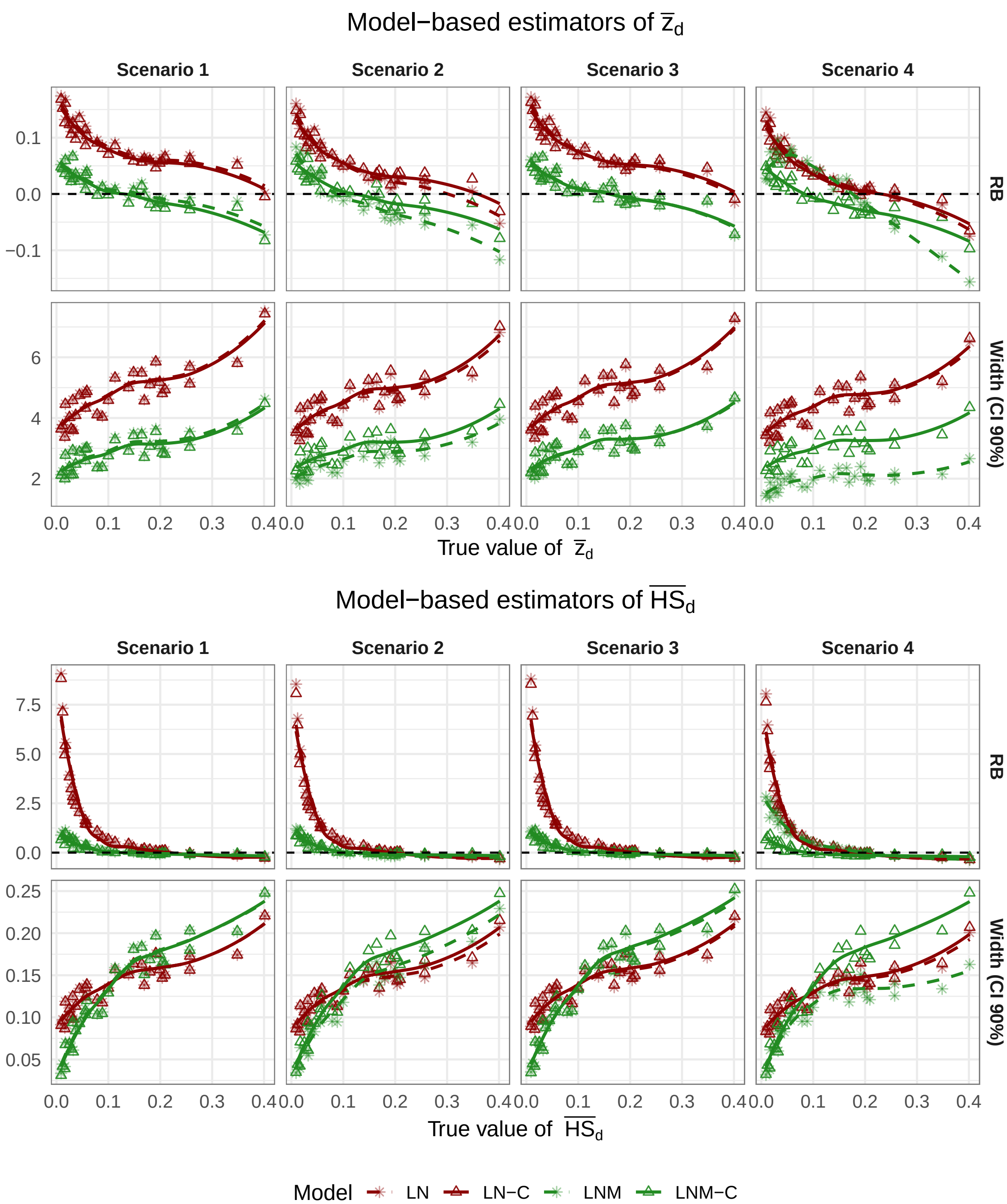


Figure S1: Relative Bias and Width for model-based estimators of $\bar{z}_d$ and $\bar{HS}_d$.

| Indicator | Dir | Dir_heap_S1 | Dir_heap_S2 | Dir_heap_S3 | Dir_heap_S4 |
|---|---|---|---|---|---|
| | | Estimators of $\bar{z}_d$ | | | |
| ARB | 0.000 | -0.025 | -0.026 | -0.021 | -0.023 |
| ARRMSE | 0.093 | 0.093 | 0.095 | 0.092 | 0.096 |
| ACov. | 0.900 | 0.901 | 0.901 | 0.903 | 0.900 |
| AAW | 3.724 | 3.747 | 3.828 | 3.704 | 3.854 |
| | | Estimators of $\overline{HS}_d$ | | | |
| ARB | -0.006 | 0.258 | 1.030 | 0.610 | 1.931 |
| ARRMSE | 0.715 | 0.859 | 1.492 | 1.132 | 2.334 |
| ACov | 0.911 | 0.914 | 0.909 | 0.894 | 0.935 |
| AAW | 0.174 | 0.204 | 0.359 | 0.259 | 0.514 |

Table S1: Average measures of performance for direct estimators of $\bar{z}_d$ and $\bar{HS}_d$.

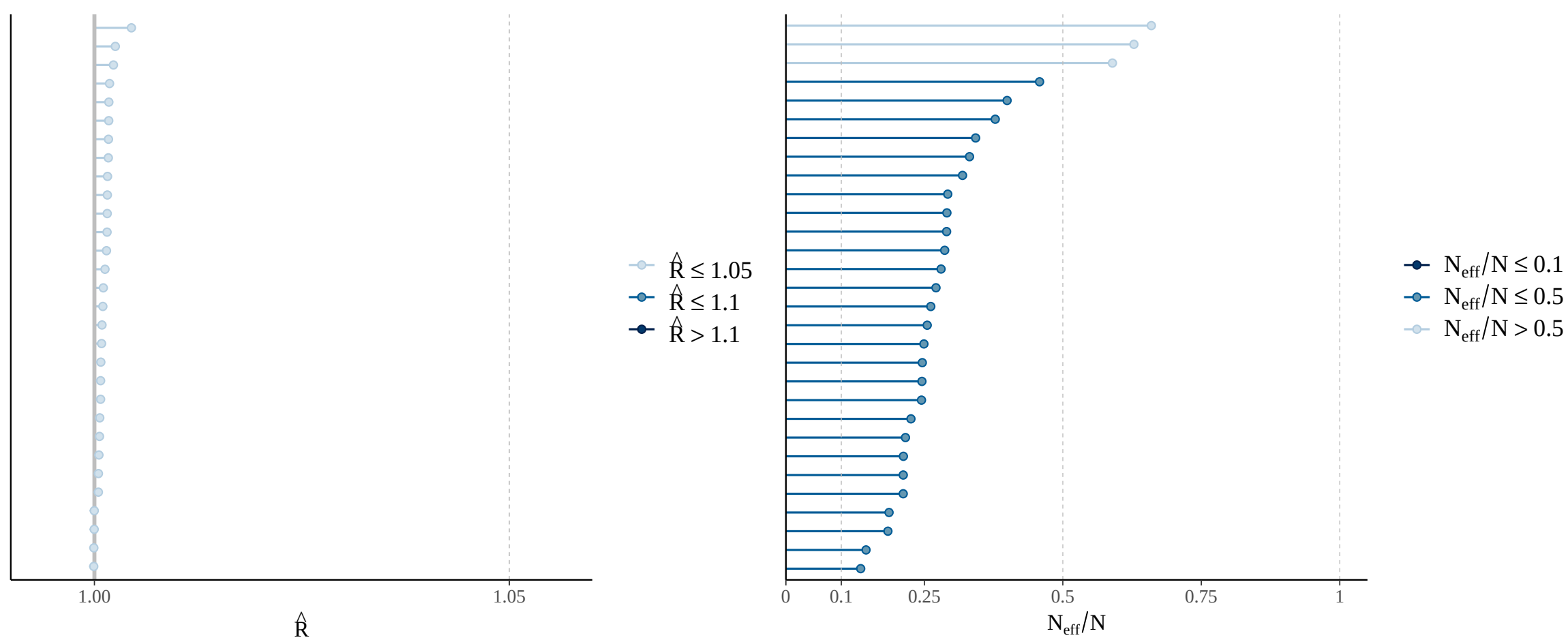


Figure S2: MCMC convergence diagnostics: values of the $\hat{R}$ statistic (left panel) and ratios of the effective sample size to the total number of posterior draws (right panel).

# S4 Additional application results

Figure S2 reports the values of the $\hat{R}$ statistic (Gelman–Rubin diagnostic) and the ratios of the effective sample size to the total number of posterior draws for the main model parameters ($\beta_0^\nu$, $\boldsymbol{\beta}^\nu$, $\beta_0^\pi$, $\boldsymbol{\beta}^\pi$, $\beta_{01}^\mu$, $\beta_{02}^\mu$, $\boldsymbol{\beta}^\mu$, $\sigma_1^2$, and $\sigma_2^2$), considering model LNM-C. The former diagnostic assesses the mixing of the four chains and should take values close to 1, a condition comfortably satisfied for all parameters. The latter measures MCMC efficiency by quantifying the effective amount of information in the posterior draws after accounting for within-chain autocorrelation. For some parameters, particularly $\beta_0^\nu$ and $\beta_{02}^\mu$, this ratio is below 0.15; nevertheless, the corresponding effective sample sizes remain greater than 1,000.

Figure S3 reports posterior predictive checks for the reported cigarette counts under alternative coarsening assumptions. Models explicitly accounting for coarsening at 1, 5 and 10 better reproduce the observed concentration of counts at focal values, indicating

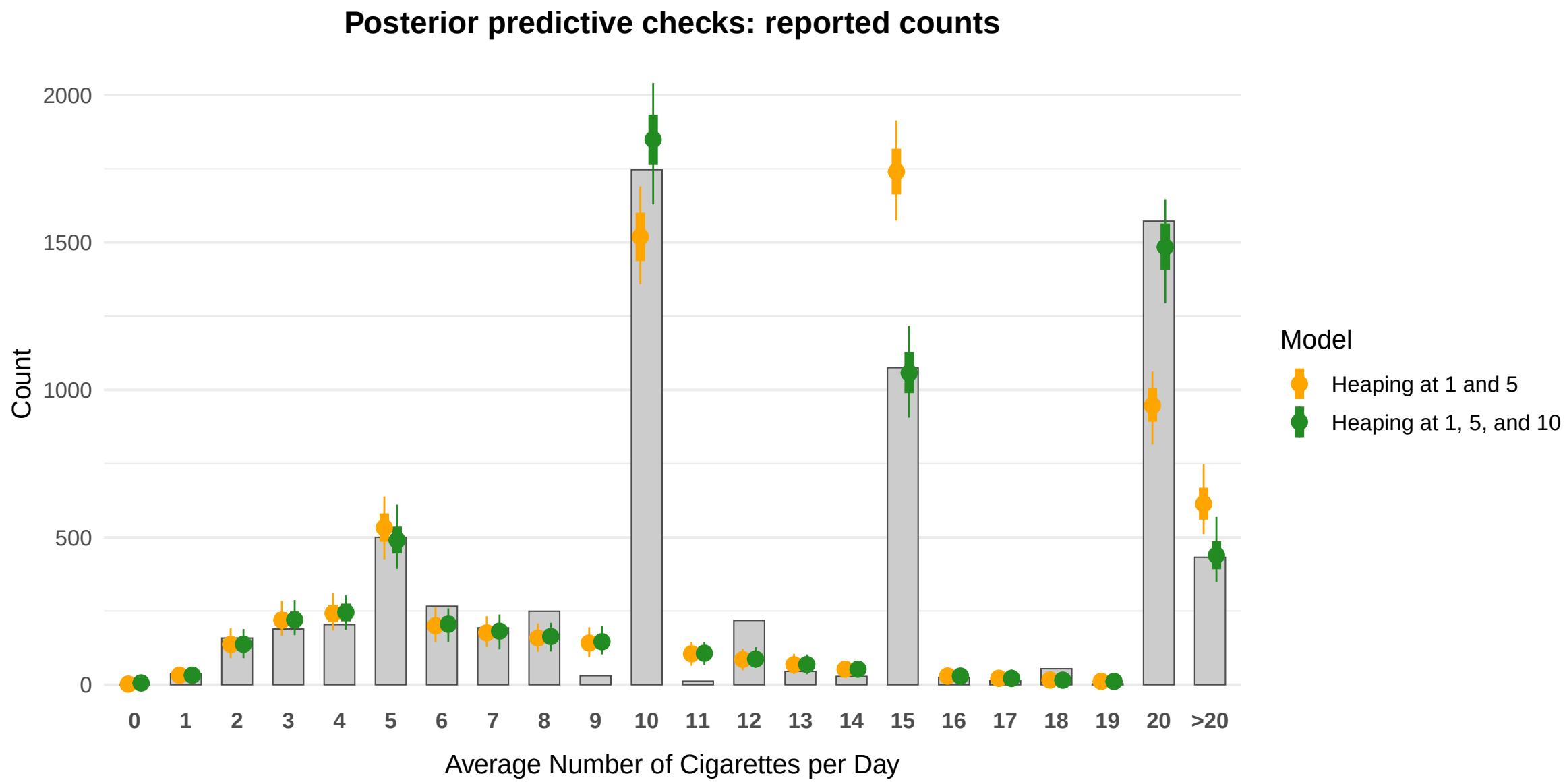


Figure S3: Frequency distribution of $Z^{\star}$ (gray bars) compared with the distribution of predicted counts under the LNM-C model with two different sets of heaping digits.

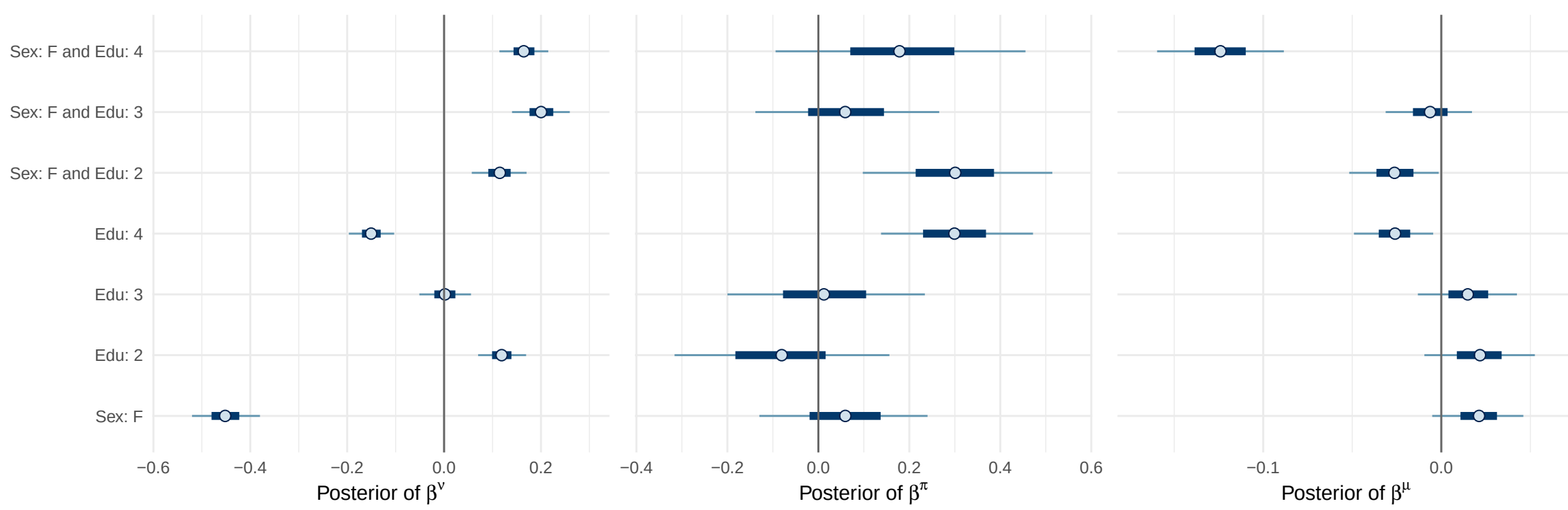


Figure S4: Posterior summaries of regression coefficients under the LNM-C model. Dots represent the posterior mean, segments report 50% (bold) and 90% credible intervals.

an improved fit to the empirical distribution.

Figure S4 displays posterior means and 90% credible intervals of the regression coefficients for the different model components. The estimates highlight heterogeneous effects of sex and education across parameters, with credible intervals providing evidence on their statistical relevance.

Figure S5 reports the parallel plots of region–age class patterns of the estimated $\overline{w}_d$ and $\bar{H}S_d$, standardized to facilitate comparison.

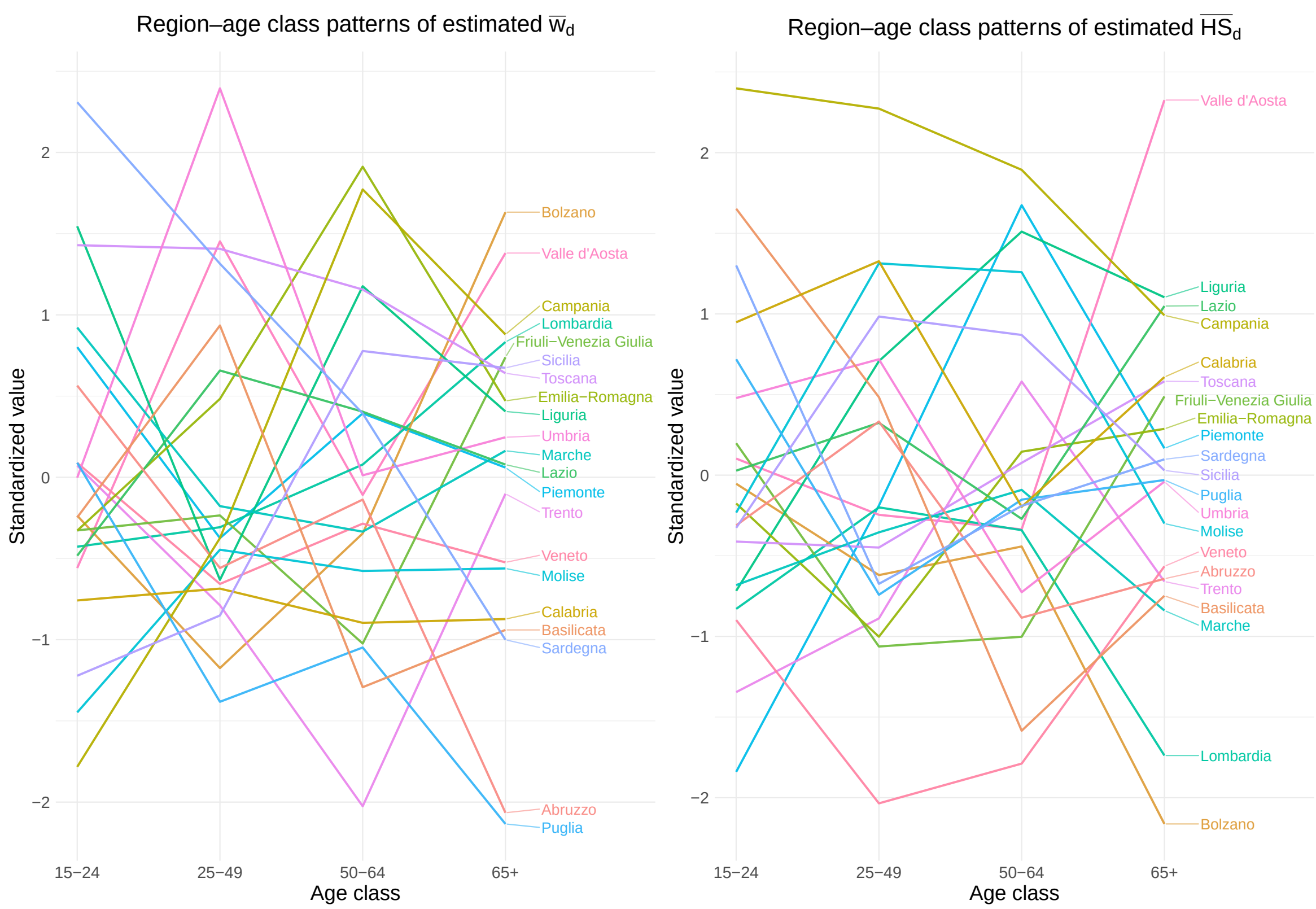


Figure S5: Parallel plots of standardized values of the estimated $\overline{w}_d$ and $\bar{H}S_d$ at the regional level across age classes.

## References

A. Gardini, E. Fabrizi, and C. Trivisano. Poverty and inequality mapping based on a unit-level log-normal mixture model. *Journal of the Royal Statistical Society Series A: Statistics in Society*, 185:2073–2096, 2022.

C. Wu and M. E. Thompson. *Sampling theory and practice*. Springer, New York, USA, 2020.
